# A formative evaluation of a brief intervention on meaning-making in the re-entry phase after curative cancer treatment

**DOI:** 10.1017/S1478951525101181

**Published:** 2026-01-12

**Authors:** Anna Visser, Lenneke Post, Joost Dekker, Lia van Zuylen, Inge Konings

**Affiliations:** 1 Department of Medical Oncology, Amsterdam UMC, Location VUmc, Amsterdam, The Netherlands; 2 Cancer Center Amsterdam, Cancer Treatment and Quality of Life, Amsterdam, The Netherlands; 3 Department of Spiritual Care, Amsterdam UMC, Location VUmc, Amsterdam, The Netherlands; 4 Faculty of Religion and Theology, VU University, Amsterdam, The Netherlands; 5 Department of Psychiatry, Amsterdam UMC, Location VUmc, Amsterdam, The Netherlands; 6 Mental Health Program, Amsterdam Public Health, Amsterdam, The Netherlands

**Keywords:** Re-entry phase, existential concerns, meaning-making, sources of meaning, intervention

## Abstract

**Objectives:**

Patients in the re-entry phase (that is, the first 18 months after curative cancer treatment) may use meaning-making to deal with existential concerns imposed by cancer and related changes in life. The purpose of the current study was to conduct a formative evaluation of an intervention aimed at supporting patients’ meaning-making process and motivating them to pick up life during the re-entry phase.

**Methods:**

Patients were included after finishing systemic treatment for breast cancer or melanoma. The intervention comprised a single one-hour conversation guided by a spiritual counselor who explored patients’ sources of meaning, in order to support them in dealing with existential concerns and changes in life in the re-entry phase. The evaluation included semi-structured interviews concerning the intervention and questionnaires assessing mental adjustment to cancer, psycho-spiritual wellbeing and meaning in life.

**Results:**

Qualitative interviews with 14 participants demonstrated an overall positive experience and appreciation of the intervention. Patients reported several benefits: reflection on existential concerns and sources of meaning, validation of sources of meaning, insights regarding the use of sources of meaning, and motivation to pick up life; and to a lesser extent: prioritizing, identifying meaningful goals, or undertaking specific action. Patients made suggestions on how to tailor the intervention more to their needs. Quantitative data showed increases on the subscales autonomy, goal-orientedness, and fairness of life with small effect sizes.

**Significance of the results:**

This study showed that an intervention to support patients with breast cancer or melanoma in the process of meaning-making in the re-entry phase after systemic treatment was positively experienced and well appreciated. It supported meaning-making, particularly through reflection on, validation and utilization of sources of meaning, and supporting motivation to pick up life. The results of the current study can be used to optimize the intervention, which can be further evaluated in a multicenter study.

## Introduction

Over the last decades, the incidence and survival rates of cancer have increased, and therewith the number of patients who have received curative treatment for cancer (Rowland and Bellizzi 2008; Arnold et al. 2019; Sung et al. 2021). After completion of treatment, patients face a transitional period known as the re-entry phase (Stanton 2012). In this phase – typically the first 18 months after curative systemic treatment, patients need to resume their former lives despite changes in life due to long-lasting physical and psychological symptoms of the cancer treatment. These difficulties are not always understood by their social environment (Tan et al. 2014). Patients face existential concerns such as fear of death, loss of control, vulnerability, isolation, rejection, meaninglessness, and threats to self-identity (Henoch and Danielson 2009; van der Spek et al. 2013; Post et al. 2020).

Especially, patients treated for breast cancer or melanoma are both groups that frequently report existential concerns in the re-entry phase (Allen et al. 2009; Drageset et al. 2016; Martínez Arroyo et al. 2019; Visser et al. 2022). After finishing long-lasting (neo-)adjuvant systemic treatments (Bleicher 2018; Seth et al. 2023; Tolaney et al. 2023), patients lose the safety net of hospital care (Holland and Reznik 2005; Stanton 2012). During the first year after finishing treatment, unmet needs are more prevalent than in long-term survivorship (Martínez Arroyo et al. 2019). These unmet needs encompass, among others, managing the expectations of being labeled a “cancer survivor,” acknowledging the impact cancer has made, and navigating life decisions (Martínez Arroyo et al. 2019). Addressing these existential unmet needs is crucial in preventing the onset of chronic and persistent distress, which can lead to diminished overall well-being (Martínez Arroyo et al. 2019).

Several effective interventions aimed at enhancing a sense of meaning and purpose have been developed in order to increase cancer survivors’ well-being. The fundamental motivational force for wellbeing, according to Frankl (1992), the founder of logotherapy, is man’s search for meaning. Informed by Frankl’s work, Breitbart et al. (2015) developed Meaning-Centered Psychotherapy (MCP), a psychotherapeutic intervention to support patients with cancer in finding meaning and purpose. Van der Spek et al. (2017) subsequently adapted MCP for cancer survivors. An alternative approach entails the enhancement of narrative meaning-making (Hartog et al. 2020), drawing upon Ricoeur’s theoretical perspective, which facilitates individuals in constructing meaning from their cancer experience through processes of storytelling and retrospective life review. An example is the spiritual life review intervention for cancer survivors (Post et al. 2020).

Still another conceptual framework is the meaning-making model of Park (2010), which conceptualizes the meaning-making process as the reappraisal of a negative event that conflicts with one’s global meaning (i.e., one’s beliefs, self-identity, goals, and values). This can result in “meanings made,” reflecting either an adapted understanding of the event or a changed (global) meaning. Such meaning-making is ultimately valuable in promoting growth and identifying meaningful goals to pursue (Park 2022). In rehabilitation medicine, an intervention was developed informed by this model, in which patients’ global meaning was explored as a starting point for setting rehabilitation goals (Dekker et al. 2020). This approach showed that addressing global meaning contributed to the intrinsic rehabilitation motivation of patients (Littooij et al. 2021).

Drawing on these promising findings and taking feasibility into account, we developed an existential intervention in a single one-hour format based on this approach. Our aim was to support patients treated for breast cancer or melanoma in their meaning-making process, with a focus on setting meaningful goals in the re-entry phase. Although existential interventions such as MCP and spiritual life review have shown promising results (Breitbart et al. 2015; Van der Spek et al. 2017; Bauereiß et al. 2018; Post et al. 2020), these interventions comprise several sessions, are solely intended for patients with high levels of (psychological) distress, and are not specifically designed for the re-entry phase (Vehling and Philipp 2018).

An intervention in the re-entry is of importance, as the transition from active treatment to survivorship can bring about a significant shift in life’s meaning (Park et al. 2024), and the meaning-making process shifts from coping with the effects of the disease and treatment to navigating the challenges of picking up life (Visser et al. 2022). Patients treated for melanoma or breast cancer reported drawing on existing sources of meaning while also adapting, strengthening, or seeking (new) sources of meaning to address existential concerns and life changes during this transition (Visser et al. 2024). We expect that enhancing patients’ awareness of their personal sources of meaning may strengthen the process of meaning-making, goal-setting, and dealing with existential concerns in the re-entry phase. Ultimately, the aim of the intervention is to empower and motivate patients to pick up life (Visser et al. 2024). The present study employed a mixed-method formative evaluation to assess the intervention, recognizing that this is crucial for ensuring effective translation into meaningful patient care (Elwy et al. 2008).

## Methods

### Study design and setting

This study used a formative evaluation method with a mixed-methods design to evaluate an intervention to support patients with their meaning-making process, with a focus on picking up life and setting meaningful goals in the re-entry phase. We used a combination of semi-structured interviews and questionnaires. The study was performed at the outpatient clinic of Medical Oncology in the Amsterdam UMC, location VUmc, between June 2021 and February 2022. This study is reported according to the guidelines for Good Reporting of A Mixed Methods Study (O’cathain et al. 2008).

### Patient selection

Eligible patients were recruited by their oncologist or nurse practitioner. Patients were included after finishing systemic treatment with curative intent for breast cancer stage or melanoma (i.e, breast cancer stage I–III and melanoma stage III). Patients had to be in the re-entry phase, defined as the period from completion of surgical and systemic treatment (with the exception of hormonal therapy) up to 18 months, with no signs of recurrence of disease. Other inclusion criteria were a minimum age of 18 years and proficiency in the Dutch language. We hypothesized that 10 to 15 interviews would be sufficient to achieve saturation, given the specificity of the phenomenon under study.

### Intervention

The intervention consisted of a single semi-structured conversation concerning meaning-making for one hour. The intervention took place in the hospital and was carried out by an experienced spiritual counselor (LP). The aim of the intervention, which was based on the results of a focus group study about meaning-making in the re-entry phase (Visser et al. 2024), was to support patients’ meaning-making process in the re-entry phase by exploring their sources of meaning. This was supposed to help patients identify the sources of meaning they can rely on during the re-entry phase and help them find new or adapted sources of meaning to deal with the changes in life and existential concerns.

During the intervention, patients were asked about their goals or challenges regarding the re-entry phase. Second, sources of meaning were explored, concerning relationships, inner posture, worldview, identity, and values in life. Finally, the spiritual counselor explored together with the patient how the patient’s sources of meaning could be helpful in dealing with their challenges in the re-entry phase and set meaningful goals for the re-entry phase. The structure of the intervention is shown in Appendix 1.

To ascertain whether patients would derive benefit from a summary of the conversation, we randomly assigned one-third of the patients to receive a summary of the conversation made by the spiritual counselor, one-third to receive a handout immediately following the conversation, which outlined the global sources of meaning (relationships, inner posture, worldview, identity and values in life), and one-third to receive no additional material to take home.

### Ethics

The present study was approved by the Medical Ethics Committee of Amsterdam UMC, location VUmc, The Netherlands. It was declared that the Research Involving Human Subjects Act did not apply to this study. All patients provided informed consent. This study was performed in line with the principles of the Declaration of Helsinki.

### Data collection

#### Qualitative data

The qualitative evaluation took place three months after the intervention. A semi-structured interview was conducted via Zoom by a trained researcher (AV). The interview protocol focused on evaluating the intervention in terms of experience and appreciation, as well as assessing the perceived benefits. Each interview lasted approximately 30–45 minutes. With participants’ permission, interviews were recorded and transcribed verbatim by a professional company.

#### Quantitative data

Patients were asked to complete a questionnaire online at baseline (T0), one week (T1), and three months post-intervention (T2). Questionnaires were completed online using the web-based survey tool Survalyzer Essential 2020. The last questionnaire had to be completed before the evaluation interview took place. The process and outcomes of meaning-making were evaluated by the Dutch version of Ryff’s Scales of Psychospiritual Well-Being (consisting of eight subscales: positive relations, autonomy, environmental mastery, personal growth, purpose in life, self-acceptance, inner strength, and relationship with a higher power) (van Dierendonck 2004) and the Personal Meaning Profile (a total score of meaning in life with five subscales: goal-orientedness, fairness in life, relationship with God, relationships with others, and dedication to life) (Jaarsma et al. 2007). The Mental Adjustment to Cancer (MAC) scale (consisting of two summary scales: positive and negative adjustment to cancer (Watson et al. 1988; Braeken et al. 2010) was used because it is known that successfully adapting or creating new meaning from the cancer experience leads to better adjustment to cancer (Park et al. 2008).

### Data analysis

#### Qualitative data

The interviews were transcribed verbatim. MAXQDA 2022 (VERBI Software 2021) was used for coding. Transcripts were analyzed using reflexive thematic content analysis (Clarke and Braun 2017; Braun and Clarke 2019). The analysis was guided by both predetermined evaluation criteria (expectations, experience, appreciation, benefits, enhancements, and recommendations) and researchers’ openness to emerging concepts. The recordings of each interview were coded by one researcher (AV); spot checks were conducted every third transcript by a second researcher (LP). The aim was to reach consensus on the outcomes by mutual agreement. After this discussion, the researchers compared, contrasted, refined, and grouped all codes into themes, after which saturation of all generated themes was evaluated. Themes were further refined as the data were sifted through multiple times. The number of patients who referenced a specific theme was quantified. This was incorporated into the text through the use of descriptors, whereby “a single patient” indicated one patient, “a few” indicated up to a quarter of the patients, “some” indicated up to half of the patients, “many” indicated up to three-quarters, and “most” indicated more than three-quarters of the patients.

#### Quantitative data

Survey data from the questionnaire survey saved in Survalyzer were entered into SPSS (IBM SPSS Statistics version 28.0, 2021). Changes in outcome measures over time were assessed using linear mixed models analysis (LMM). A fixed effect for measurement-time and a random effect for subjects were used. Effect sizes were calculated by dividing the difference in change since the baseline by the pooled standard deviation, at the separate points in time (one week and three months post-intervention). Effect sizes of 0.2 were categorized as small, 0.5 as moderate, and 0.8 as large (Cohen 2013). Only effect sizes were reported, because it is well recognized that effect size is more informative than statistical significance in small samples, as *P*-values are strongly influenced by sample size (Sullivan and Feinn 2012).

## Results

### Patients

A total of 18 eligible patients were asked to participate. One participant refused without providing a reason, one patient did not feel the need to participate, and another patient felt the intervention was too soon after the end of treatment. Fifteen patients were included. Seven patients had been treated for breast cancer, and eight patients had been treated for melanoma. Participant demographics are presented in Table 1.

**Table 1.** Demographics of participants

	Patients treated for melanoma (n = 8)	Patients treated for breast cancer (n = 7)	Total (n = 15)
Gender			
*Female*	3	7	10
*Male*	5	–	5
Age, mean (range)	60.6 (range 45–85)	50.6 (range 33−62)	55.9 (range 33−85)
Diseases stage			
*Stage I*	–	1	
*Stage II*	–	3	
*Stage III*	8	3	
Treatment			
*Surgery*	8	7	
*Immunotherapy*	8	0	
*Chemotherapy*	–	7	
*Radiotherapy*	–	5	
*Hormonal therapy*	–	2	
Months after completion of treatment, mean (range)	1.6 (0−3)	1.7 (0−3)	1.7 (0−3)
*Number of patients living with a partner*	8	7	15
*Number of patients who have children*	8	7	15
Education			
*University*	1	1	2
*Higher professional*	2	4	6
*Secondary vocational*	1	0	1
*Unknown*	4	2	6

### Study adherence

**Figure 1.**
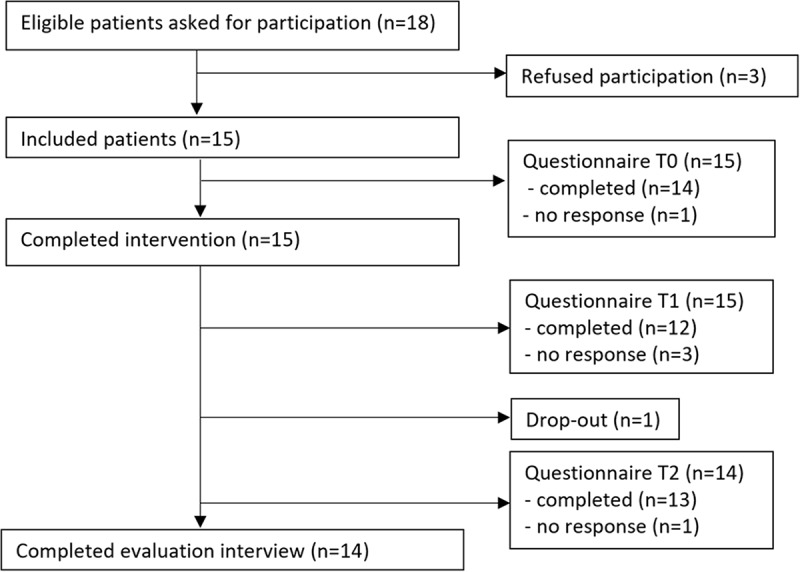
Flow diagram of the inclusion and data collection.

One patient with melanoma withdrew from the study without providing a reason after completing the questionnaire at baseline, the intervention, and the questionnaire one week after the intervention. Consequently, 14 patients completed the evaluation interview. Of the 15 patients, 11 completed the questionnaire at all three time points. See Figure 1.

### Qualitative results

The following themes resulted from the analysis:

A detailed description of each theme and subtheme is provided, accompanied by quotations from the evaluation interviews in Table 2.

**Table 2.** Themes with description of each subtheme accompanied with quotes

	Description	Featured quotes
Theme 1: Overall evaluation		
Experience	Most patients described the conversation as positive, pleasant, good, relaxed, enlightening, and valuable.	*For me, it was a beautiful conversation to have* (33-year-old woman treated for breast cancer) *I left with a pleasant feeling (*45-year-old man treated for melanoma).
Appreciation	Most patients appreciated the opportunity to share their experiences with someone outside their immediate circle, allowing them to express themselves freely. Many patients would recommend it to others. Some patients mentioned they appreciated the aftercare and support after treatment. The intervention was rated with 8.3 out of 10 [range 7−10].	*You have to re-frame your life. It sounds a bit weird (…), but it just is. So I think it is really valuable to have a different kind of aftercare. Not from the oncologist, but like, how are you doing, and what are you running into?* (56-year-old female treated for breast cancer).
Evaluation of the discussed sources of meaning	Many patients were positive about the sources of meaning that were discussed during the intervention.	*It is kind of the topics that matter (*62-year-old man treated for melanoma).
	Worldview, in particular religion, was the least relevant for most patients. A few patients felt it was difficult to talk about sources of meaning; however, because they felt at ease, it was possible to do so.	*Sometimes [I found it] also quite complicated, because you suddenly have to think about things you’ve got to express yourself about, and I’m not very strong at that, but it went well. (*60-year-old female treated for melanoma).

Theme 2: Perceived benefits		
Reflection on existential concerns and sources of meaning	Many patients reported that the intervention helped them reflect on their past experiences, their sources of meaning, and the future.	*[The conversation helps] to go through things in a different way and to stop again and actually look back* (45-year-old man treated for melanoma). *The questions that she asked made me reflect (…) what it was like for your family and who supported you?* (56-year-old woman treated for breast cancer).
	The use of clear and probing questions was positively noted because this helped the reflection process. Also, the intervention helped to address existential concerns.	*I think the conversation helped me think about certain issues, like: how do I see the future? Am I still scared or not?* (72-year-old woman treated for melanoma).
Validation of sources of meaning	Most patients reported that their sources of meaning were (re)confirmed during the intervention.	*I’m not making up how I position myself in it [in life], but it’s also confirmed* (51-year-old woman treated for melanoma).
	Sources of meaning became clearer and were validated .	*It’s nice to have a conversation like that where you are thinking: oh yeah, but wait, what’s really important to me? And that’s what I learned from that conversation: that I just have a very clear list of what’s important to me. It [the list] is something I can refer to in the future.* (33-year-old woman treated for breast cancer).
Insights regarding the use of sources of meaning	Many patients reported that the intervention gave insight into how they can use their existing sources of meaning.	*[It gave] insight into myself, how I function, how others see me. (…) And it’s kind of funny to think about that. Not something you do every day, of course. So yeah, I think I learned a few things, including how I am in life and how I deal with it.* (72-year-old woman treated for melanoma).
	Or they got insight into adapted, strengthened, or new sources of meaning.	*Sometimes I think, oh, I have to do this, and I have to do that, and then also because of such a conversation, you find out: you don’t have to do it all. You know, you can also go and seek solutions for that, for example, with family or friends. So you get a little reassurance that life is not like before, actually.* (48-year-old female treated for breast cancer).
	Patients also realized how their sources of meaning could help with certain existential concerns.	*You can dwell on it [that my friends were not there for me], but that’s negative. So it’s better to put it aside. (..) And during this conversation, it became clear [that I am down-to-earth and able to relativize]. So that allows me to give it a place.’* (51-year-old man treated for melanoma).
Motivation to pick up life	Many patients reported that the intervention motivated them to pick up life again and gave them support to pick up life.	*It’s a boost, a push like we’re moving on again.* (85-year-old man treated for melanoma). *It did give me the realization that, because you’ve connected a few dots and you do get some kind of confirmation, you can do it. It’s okay also to feel bad every now and then. It [the intervention] did do me good. I’ll say that. I was all happy-go-lucky when I walked out.* (46-year-old woman treated for breast cancer).
	Thus, the intervention made patients focus on the future again, instead of looking back or thinking about their disease.	*More like [the realization] OK, it [this period] is really done now. I have to start thinking about the future again.* (56-year-old woman treated for breast cancer).
Setting priorities (in picking up life)	For some patients, the intervention helped to get their priorities straight.	*What I got out of it was what is important to me, what do I want to focus on, and how do I want to use that* (48-year-old woman treated for breast cancer).
	Prioritizing helped them with picking up life.	*It gives you tools, like where to start and to accept that you can’t start everywhere anymore. Work was always a big part of my identity before I got ill. (…) When you really look at what is meaningful in your life (…), you find out that work really only comes in at number 5 or so. It makes it easier for you to accept that you don’t start with that anymore* (33-year-old woman treated for breast cancer).
Identifying meaningful goals	Some patients identified meaningful goals during the intervention, although they did not refer to them as goals.	*[The most important thing during the interview turned out to be] how I’m dealing with the diagnosis and what I’m going to do afterwards, and what I want my attitude to be like (72-year-old* female treated for melanoma).
	Meaningful goals could be values to live by, what to strive for in life, or maintaining things in life that they identified as important. Patients who had already set goals for themselves said that the intervention helped them to be more aware of their goals in daily life.	*No new goals, but just becoming aware of those goals again, so to speak. So it is a conversation I still think about every now and then because, yes, the more often you express things and put them in order, the easier it becomes to live by them.* (33-year-old woman treated for breast cancer).
	Other patients did not set goals during the conversation; some indicated that they would require a follow-up intervention in order to be able to set goals, others generally did not set goals in life and therefore did not feel the need to do so after the intervention . One patient said the intervention did not help to achieve her goals to improve her lifestyle.	*Interviewer: And did you talk about any particular goals you had during the interview? Patient: Yes, she asked about that. But that… I always see what comes my way. It’s not something. I don’t have a bucket list, mind you.* (62-year-old man treated for melanoma).
Undertaking specific action post-intervention	Some patients gave an example of how they changed something in their behaviour after the intervention	*Well, what I’m doing more is that I take the other person a little less into account. I hold my tongue a little less, or so it’s called.* (51-year-old woman treated for melanoma).
	Some took specific action. However, most patients reported that they did not undertake specific action after the intervention and just continued with what they were doing. Some of them said that they had already undertaken action, regardless of the intervention.	*Interviewer: When you realized during the conversation that they are there for you and that you are grateful for them, did that still do something to you, or did you do something with it?* *Patients: Oh yes, I then bought one of those breast cancer bracelets and stuff, you know, so we all had the same bracelets.* (46-year-old woman treated for breast cancer).

Theme 3: Optimization of the intervention		
Timing of the intervention	While many patients perceived the timing of the intervention as optimal, some felt that it occurred either too early or too late.	*I think I had just finished treatment three months ago. So I was like, yes, it is a good time to have that conversation, because then your normal life starts again, you don’t have therapy anymore. So you can also reflect a bit. I just thought that was a very good moment* (56-year-old woman treated for breast cancer).
	Various factors influenced this perception, for example, one patient still received hyperbaric oxygen therapy, another patient felt she had already resumed life during adjuvant chemotherapy due to the minimal side effects.	*For me, it could have been earlier, much earlier, I think. However, that’s also because I already saw the six months of chemo pills as the start of recovery.* (33-year-old woman treated for breast cancer).
Role of the spiritual counselor	Most patients experienced the spiritual counselor as open, calm, and empathic. They reported feeling listened to and heard by the spiritual counselor.	*The conversation partner was very pleasant. She took her time and repeated things. She listened, summarised, and asked follow-up questions.* (45-year-old man treated for melanoma).
	A few had wished for more guidance, advice, or more critical responses from the spiritual counselor.	*I did most of the talking. I told how I saw it instead of getting input back, like a reflection of what I said was good or not.* (49-year-old woman treated for breast cancer).
Preparation	Some patients did not feel prepared for the intervention. For example, they indicated that “setting meaningful goals” sounded vague or that the goal of the intervention was not clear prior to taking part in the intervention. They would recommend managing expectations better in advance.	*A bit more information in advance about how such an interview goes [would be an improvement], because you actually go in completely blank. You have no idea what’s going to be asked, and if I could have prepared for that, it might have been easier. (…) Now, all of a sudden, you have to answer quite complicated questions.* (72-year-old woman treated for melanoma).
Supporting materials	Many patients are recommended to provide a summary of the conversation afterwards. Patients who got a summary of their conversation were positive about it. They saw it as an extra confirmation, check, or summary for themselves. Patients who did not receive a summary expressed a desire for one.	*It’s nice to have it clearly listed afterwards. (..) It feels a bit like a cheat sheet, where you just have points in bullet points, and you know about yourself. Look, obviously, it’s not like someone is telling you what your goal should be. However, it provides a starting point for you to express your own thoughts and emotions. So, yes, I did find it very enlightening. It’s a reminder, and that’s nice.* (33-year-old woman treated for breast cancer).

### Overall evaluation

Qualitative interviews demonstrated an overall positive experience and appreciation of the intervention. Most patients reported they felt at ease and appreciated the support and the opportunity to share their experiences. Many patients indicated that the conversation covered all important topics.

### Perceived benefits of the intervention

Patients reported the following perceived benefits of the intervention: reflection on existential concerns and sources of meaning, validation of sources of meaning, insights into the use of sources of meaning, and motivation to pick up life. Patients reported, to a lesser extent, setting priorities, identifying meaningful goals, and undertaking specific actions post-intervention.

### Optimization of the intervention

Many patients made suggestions on how to tailor the intervention more to their needs and requested additional supporting materials. This included suggestions for adjusting the timing of the intervention and the role of the spiritual counselor to better meet individual needs, as well as recommendations for more comprehensive preparation and a summary of the intervention afterwards.

### Quantitative results

Patient reported changes in the experience of meaning in life and mental adjustment to cancer following the intervention. Increases with small effect sizes were observed in the subscales fairness in life, autonomy, and goal-orientedness one week after the intervention. Goal-orientedness also showed a small effect size improvement three months after the intervention. Detailed results are presented in Appendix 2.

## Discussion

This mixed-method formative study evaluated an intervention to explore one’s sources of meaning and help set meaningful goals for the re-entry phase after systemic cancer treatment. The intervention was designed to support meaning-making after cancer treatment to help patients address existential concerns (Allen et al. 2009; Stanton 2012; van der Spek et al. 2013; Dekker et al. 2017; den Bakker et al. 2018; Park et al. 2024) and motivate patients to pick up life. Patients responded positively, reporting multiple benefits. The qualitative findings highlight the potential of a single one-hour session to facilitate patients’ meaning-making process. The intervention facilitated reflection, and it validated what patients find meaningful, as well as the meaning they ascribe to their experiences and actions. It motivated patients to pick up life again and to use identified sources of meaning to address their existential concerns. It prompted some patients to reorient in life by prioritizing, setting goals, or adopting different approaches to their situation.

Notably, the benefits varied in the degree of patient engagement. Reflection was facilitated by probing questions of the spiritual counselor, which enabled patients’ awareness and acknowledgment of their sources of meaning and existential concerns. Furthermore, the intervention instigated validation, motivation, and insights into how to use their sources of meaning during and after the intervention. This empowered patients to derive strength from sources of meaning and actively self-manage their meaning-making processes. Empowerment has been increasingly recognized as an important component in cancer survivorship care (Jørgensen et al. 2018). Some studies highlight its association with the motivation to find meaning after cancer (Mok et al. 2004) and the pursuit of personal and health-related goals (Groen et al. 2015). The most active forms of engagement were reflected in the themes of prioritization, identifying meaningful goals, and action-taking, collectively representing a form of existential reorientation. Reflection, empowerment, and re-orientation may represent conceptual constructs that require further investigation in the future to refine the intervention.

Based on the intervention on setting meaningful goals in rehabilitation (Littooij et al. 2021), we expected goal-setting for the re-entry phase to be a clear benefit. Goal-orientedness showed a small effect size improvement after intervention; however, the qualitative results regarding goal-setting were mixed. Not all patients reported setting goals, and the desire to set goals varied among patients. Moreover, some patients identified meaningful goals but were not aware of them as such. These goals were not actively and specifically targeted toward the re-entry phase, but were more personal strivings in life. Earlier research on goal-setting in cancer survivors showed that patients find it hard to specify exact goals (Hauken et al. 2014), and that goals that are set can be viewed more as general hopes or desires, such as “get back to normal” and “stay cancer free.” These goals are similar to the goals we found in our focus groups prior to the development of this intervention (Visser et al. 2024). This raises the question of whether all patients actually feel the need for goal-setting, and whether the current intervention addresses goal-setting sufficiently and appropriately. We propose that in the future design of the intervention, the spiritual counselor involved in the intervention makes implicit goals more explicit and documents them as “meaningful goals” in the summary provided to patients afterwards. Additionally, if patients wish to, the intervention could facilitate the translation of general goals into more specific actionable goals by providing patients with suggestions on how to bring this into practice (Bovend’Eerdt et al. 2009).

Our intervention is the first existential intervention that uses a single session of one hour only for patients, specifically in the re-entry phase, that can empower patients to resume their lives (Oh and Kim 2014). This approach places minimal strain on hospital staff resources. As this current evaluation is of a formative nature, the findings can be used to maximize the benefits of the intervention. First, it is essential to align the timing with the patient. The focus of the meaning-making process in the re-entry phase shifts from focusing on the disease and treatment to focusing on picking up life (Visser et al. 2024). Our findings showed that the optimal timing for the intervention was depended on patients’ perception of when they felt ready to resume their “normal life” again. It should be the responsibility of the healthcare provider to liaise with the patient to ascertain this moment and to schedule the intervention accordingly. Furthermore, not all patients felt prepared for the intervention. It is recognized that both patients and healthcare professionals lack sufficient knowledge regarding the nature of existential and spiritual interventions (van de Geer et al. 2016; Best et al. 2022; Stavig et al. 2022). Therefore, patients should be adequately informed to know what to expect, and healthcare professionals should be better informed to counsel patients. Finally, in accordance with the findings of the evaluation, a summary should be provided to patients following the intervention to enhances its benefits of the intervention.

Based on literature, we anticipated that supporting the meaning-making process would strengthen certain sources of meaning, leading to increases in meaning in life and psychospiritual well-being, as well as greater positive adjustment to cancer (Park et al. 2008; Park and Gutierrez 2013; Breitbart et al. 2015; Bauereiß et al. 2018; Visser et al. 2024). Our qualitative findings suggest that sources of meaning were strengthened, while quantitative findings demonstrated modest improvements in goal-orientedness, fairness of life, and autonomy. Notably, the quantitative analyses were constrained by the limited sample size of 11 patients who completed all three questionnaires. However, future research should also reconsider the appropriateness of the instruments used to evaluate the intervention, as the limited sample size may not be the only explanation for the modest quantitative effects. Furthermore, a more targeted approach to identify those who derive the greatest benefit from this intervention is necessary to optimize its effectiveness. A multicenter study with a larger sample size should be conducted to assess the feasibility, effects, and determine the best methods for implementing this intervention.

### Strengths and limitations

In addition to qualitatively assessing patients’ experiences, appreciation, and perceived benefits, a strength of this formative evaluation was its ability to identify opportunities to further optimize the intervention. Another positive aspect was the high participation rate. However, it should be noted that the study was susceptible to selection bias. It is possible that healthcare providers may have selected patients they believed are suitable for this type of intervention.

## Conclusion

This formative study shows that a one-hour intervention on meaning-making is applicable, positively experienced, and well appreciated by patients treated for breast cancer and melanoma in the re-entry phase after cancer. The qualitative evaluation presented promising results that a single one-hour intervention supported patients’ meaning-making process, in particular through reflection on, validation of, and use of their sources of meaning, and supporting motivation to pick up life. The evaluation identified possibilities for further optimization of the intervention. A multicenter study is needed to evaluate the feasibility, implementation, and beneficial effects of the intervention.

## Data Availability

The datasets generated during and/or analyzed during the current study are available from the corresponding author on reasonable request.

## Appendix 1:Structure of the intervention

**Table:**

**1**	**Introduction**	The purpose of the intervention is explained	5 min
**2**	**Looking ahead to the re-entry phase**	Patients’ hopes/expectations/goals/challenges for the re-entry phase are discussed	10 min
**3**	**Exploring sources of meaning**	In an open conversation, the following sources of meaning are explored:	30 min
		- Identity	
		- Relationships	
		- Core values	
		- Worldview	
		- Inner posture	
**4**	**Supporting the meaning-making process for the re-entry phase**	- Goals, challenges, and sources of meaning that were discussed are summarized	15 min
		- Which source of meaning is the most central is discussed	
		- How sources of meaning can help in picking up life is explored	
		- Meaningful goals are set	
**5**	**Close**		5 min

## Appendix 2

**Table:**

Personal meaning profile			
Dependent variable	Mean (scale 0–100)	SD	Effect size
Goal-orientedness	T0: 72.995 T1: 75.201 T2: 75.195	14.37878	**0.1534** **0.1530**
Relation with god	T0: 31.096 T1: 31.132 T2: 28.548	23.66477	0.0015 −0.1077
Dedication to life	T0: 76.508 T1: 76.224 T2: 76.411	12.59573	−0.0225 −0.0077
Fairness of life	T0: 69.929 T1: 72.943 T2: 67.964	12.37677	**0.2435** −0.1588
Relationships with other people	T0: 85.690 T1: 85.660 T2: 82.978	13.05202	−0.0023 −0.2079
Total	T0: 67.253 T1: 68.612 T2: 66.830	9.54975	0.1423 −0.0443
**Mental adjustment to cancer**			
**Dependent variable**	**Mean**	**SD**	**Effect size**
Negative adjustment	T0: 28.571 T1: 28.757 T2: 28.495	6.46239	0.0288 −0.076
Positive adjustment	T0: 50.500 T1: 51.239 T2: 49.677	6.20478	0.1191 −0.1326
**Psychospiritual well-being**			
**Dependent variable**	**Mean**	**SD**	**Effect size**
Positive relations with others	T0: 5.031 T1: 5.022 T2: 4.933	0.78602	−0.0115 −0.1247
Autonomy	T0: 4.403 T1: 4.549 T2: 4.316	0.58723	**0.2486** −0.1482
Environmental mastery	T0: 4.776 T1: 4.752 T2: 4.595	0.64834	−0.0370 −0.2792
Personal growth	T0: 4.673 T1: 4.640 T2: 4.639	0.65338	−0.0505 −0.0520
Purpose in life	T0: 4.653 T1: 4.712 T2: 4.578	0.58272	0.1012 −0.1287
Self-acceptance	T0: 4.566 T1: 4.561 T2: 4.517	0.52579	−0.0095 −0.0932
Inner strength	T0: 4.636 T1: 4.534 T2: 4.497	0.70551	−0.1446 −0.1970
Relationship with a higher power	T0: 2.804 T1: 2.707 T2: 2.833	1.48610	−0.0653 0.0195
